# Study of the Stability of Citrate Capped AgNPs in Several Environmental Water Matrices by Asymmetrical Flow Field Flow Fractionation

**DOI:** 10.3390/nano11040926

**Published:** 2021-04-05

**Authors:** Aaron Boughbina-Portolés, Lorenzo Sanjuan-Navarro, Yolanda Moliner-Martínez, Pilar Campíns-Falcó

**Affiliations:** MINTOTA Research Group, Departament de Química Analítica, Facultat de Química, Universitat de Valencia, 46100 Burjassot, Spain; abough@alumni.uv.es (A.B.-P.); lorenzo.sanjuan@uv.es (L.S.-N.); yolanda.moliner@uv.es (Y.M.-M.)

**Keywords:** silver nanoparticles, citrate capping, potable water, transitional water, sea water, aggregation, AF4

## Abstract

Asymmetrical flow field-flow fractionation (AF4) coupled to UV-Vis and dynamic light scattering (DLS) detectors in series, was tested for stability studies of dispersions of citrate-capped silver nanoparticles (AgNPs) in several water matrices. The main goal is to provide knowledge to understand their possible behavior in the environment for short times since mixturing (up to 180 min). Ultrapure (UPW), bottled (BW1, BW2), tap (TW), transitional (TrW) and sea water (SW) matrices were assayed. Observations were compatible with the aggregation of AgNPs, a change in the plasmon band and a size growth with time were done. Fractograms showed different evolution fingerprints in the function of the waters and batches. The aggregation rate order was BW2, SW, TrW, BW1 and TW, being BW2 the lowest and TW the highest. NP aggregation can be induced by increasing the salt concentration of the medium, however transitional and sea waters did not follow the rule. Both matrices presented a lower aggregation rate in comparison with other aqueous matrices with much lower ionic strength (BW1 and TW), which can be explained by the potential presence of dissolved organic matter and/or the high concentration of halides providing their stabilization and passivation, respectively. AF4 provides relevant information with respect to static DLS and UV-Vis Spectroscopy showing that at least two populations of aggregates with different sizes between them, depending on both, the mixture time for a given matrix and type of water matrix for the same time.

## 1. Introduction

In recent years, the use of noble nanoparticles (NPs) has been continuously increasing due to their physical and chemical properties. These properties give them unique characteristics, which have aroused great interest [1,2,3,4,5,6,7,8]. These characteristics, convert NPs, specifically AgNPs, into tools used as potential applications in a wide variety of fields [9,10,11,12,13,14,15]. Moreover, and despite the benefits that their use can generate for the diagnosis of diseases [15,16] or their treatment by acting as anticancer or antimicrobial agents [17], NPs also appear on the scene as potential emerging pollutants whose effects on human health [9,18,19] and the environment are not yet fully known [20,21,22].

Although surface properties are crucial in understanding the environmental behavior of AgNPs in terms of their stability against aggregation, mobility, and toxicity, phase transformations of AgNPs have the largest impact on their fate and also lead to modification of their surface properties [22]. The relationship between surface properties of AgNPs and their environmental behavior has only been partly explored.

Recently, the stability of citrate-coated AgNPs in natural brackish water was studied in [23] by employing UV-Vis spectroscopy, dynamic light scattering (DLS) and transmission electron microscopy (TEM). The authors established that the potential environmental risks induced by AgNPs were reduced with increasing salinity by their sedimentation and dissolution, suggesting that the order of potential risk in different natural aquatic systems probably varied as follows: freshwater > brackish water > seawater.

Here, a study for evaluating the stability of citrated-capped silver nanoparticles (AgNPs) in different water matrices was performed considering that citrate is the most used capping agent. There is growing evidence that small nanoparticles (i.e., <20–30 nm core) can display properties significantly different relative to larger nanoparticles or bulk materials of the same composition [22]. In order to achieve this goal, various suspensions were prepared using different aqueous matrices, and their evolution over time was followed by UV-Vis spectroscopy and asymmetric flow field-flow fractionation (AF4) with UV-Vis and dynamic light scattering (DLS) detectors in series. Parameters that influence stability are discussed; it is well known that metallic Ag is not thermodynamically stable under most environmental conditions. Besides in particular, aggregation, agglomeration, dissolution, reaction, or replacement of surface capping are potential transformations that can alter the performance and stability of metallic NPs.

Capillary liquid chromatography (Cap-LC) [23,24,25], ICP-MS [26,27], exclusion size chromatography [28,29] or capillary electrophoresis [30,31] were used for studying dispersions of NPs too. Cervantes-Aviles et al. in [32] used nanoparticle tracking analysis (NTA), single particle ICP-MS (sp-ICP-MS), and localized surface plasmon resonance (LSPR) analysis to study the stability and dissolution of polyvinylpyrrolidone coated AgNPs at µg·L^−1^ levels in synthetic wastewater (SWW).

Asymmetrical flow field-flow fractionation (AF4) is a separation technique for particles and macromolecules [33,34]. A cross-flow (hydraulic pressure gradient field) perpendicular to the channel is controlling separation. Separation is based on size (hydrodynamic radius) and diffusion back to the region of lower size acts as a counteracting force. A steady-state distribution of analyte is established with the highest size at the accumulation wall that decreases toward the center of the channel [34].

In a previous paper [35], our group studied the dilution-induced variation with time (up to 72 h) of citrate capped AuNPs and AgNPs of several sizes with ultrapure water by AF4 coupled with UV-Vis and DLS detectors. Absorbance signal in diluted dispersions decreased as a function of time and particle size being dissolution the process due to hydrodynamic diameter was constant. In this work, AF4-UV-Vis-DLS was used to characterize and analyze citrate capped AgNPs of 20 nm core size focusing on their performance and stability in the function of time for short times (up to 180 min) by dilution of them in several environmental water matrices. The main goal is to provide knowledge to understand their possible behavior in the environment.

## 2. Materials and Methods

### 2.1. Materials

Commercial aqueous AgNP dispersions of 20 nm (size determined by transmission electron microscopy, TEM), 20 mg·L^−1^ with sodium citrate as stabilizer, were provided by Sigma Aldrich ( Saint Louis, MO, USA). AF4-UV-Vis-DLS liquid carrier was prepared with sodium azide 0.02% (NaN_3_, Panreac, Castellar del Vallés, Barcelona, Spain). Methanol (VWR, Radnor, PA, USA) was used for cleaning AF4 system.

Ultrapure water was obtained using a Sybron Barnstead NANOpure II water purification system (ρ = 18.2 MΩ cm; Total organic carbon <2 ppb).

### 2.2. Instrumentation

UV-Vis spectra were obtained by using a Varian Cary 60 UV-Vis spectrophotometer (Agilent Technologies, Santa Clara, CA, USA). The analytical signal was recorded between 300 and 600 nm. Conductivity, pH and potential were measured with a Hach sensION+MM150 DL portable multimeter (Düsseldorf, Germany).

An optical microscope (ECLIPSE E200LED MV Series, Nikon Corporation, Tokyo, Japan) was employed under bright-field illumination using 10× and 50× objectives to show the aggregated AgNPs. Nis Elements 4.20.02 software (Nikon Corporation, NY, USA) was used for acquiring the images.

AF4 measurements were carried out by using an AF2000 MT model purchased from Postnova Analytics Inc. ((Landsberg am Lech, Germany). The channel was 29 cm long with a 10 kDa regenerated cellulose membrane and 350 μm channel spacer. The flows were provided by two separate pumps and the cross-flow was obtained by a separate piston pump, which was constantly adjusted. The liquid carrier was high purity Mili-Q water containing 0.02% sodium azide. Optimal separation was achieved using the following conditions given in Table 1:

AF4 system was online coupled with a DLS detector (which can work off-line too) with temperature control (Nano-ZS, Malvern, UK), and UV-Vis detector (SPD-20AV, Postnova, UK), which was operated at the wavelength of 395 and 750 nm.

### 2.3. Analysis of Dispersions of AgNPs Diluted with Different Aqueous Matrices

Four commercial batches of citrate-capped AgNPs with the same nominal concentration given by the supplier and different caducity data (March and October 2020, named M and O, respectively) were tested. Dispersions were measured at several times from the preparation by using both, the AF4-UV-Vis-DLS system and the UV-Vis spectroscopy. For some of them, the off-line DLS, and the optic microscopy were also employed to study the performance of the AgNPs in different aqueous matrices. Suspensions of the different commercial batches were prepared using different types of diluent: ultrapure (UPW), bottled (BW1 and BW2), tap (TW), transitional (TrW) and sea water (SW). A dilution of 1/4 of the bulk dispersion with given environmental water was prepared in all cases and the time 0 corresponding to the time of the mixture preparation. Two replicates were carried out in all assays.

## 3. Results and Discussion

### 3.1. Characterization of Several Batches of Citrate Capped AgNPs

**Figure 1:**
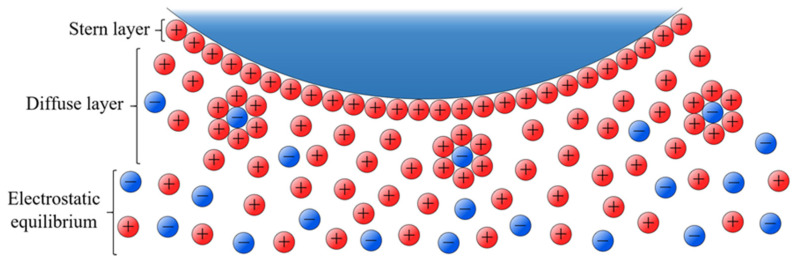
Schematic representation of the electrical double layer in a negatively charged NP.

As for the electrostatically stabilized NPs, the fact of having charged species in the capping induces changes in their environment. A tightly bonded layer known as the Stern layer is formed due to the negatively charged surface of the AgNPs attracts some of the positive ions (counter ions) in the dispersion. Stern layer gives rise to a diffuse region of positive ions, shielded and solvated negative ions and dispersant molecules, known as the diffuse layer, which extends from the Stern layer to electrostatic equilibrium. All this set of charged species distributed around the NP forms what is known as the electric double layer (see Figure 1). This electrostatic envelope protects the NPs and prevents them for aggregation. The DLVO theory (from Derjaguin, B.; Landau, L.; Verwey, E.; Overbeek, T.) [36] is capable of explaining the stability and behavior of colloidal systems as an equilibrium between opposite forces: the attractive van der Waals forces and the electrostatic repulsion of the electric double layer. Consequently, the stability of the system depends on the resultant force of both, whose magnitude determines the kinetic energy that two NPs must have when colliding to overcome the energy barrier that prevents their aggregation. However, DLVO lacks an atomistic description of the electrical double layer at the surface of NPs, which excludes the examination of the molecular properties of the solvent electrolytes, and eventually aggregating onto the NP [37]. Franco-Ulloa et al. [37] developed a theoretical framework that determines the surface coverage of charged ligands onto spherical NPs and its application permits to describe at the molecular level the dispersion state of citrate-capped gold nanocolloids.

The fundamental interactions underlying citrate-mediated chemical stability of metal nanoparticles, and their surface characteristics dictating particle dispersion/aggregation in aqueous solutions, are largely unclear [37]. Moreover, dilution-induced changes, mainly on the surface interactions that may affect the performance of AgNPs. These changes depend on the kinetics of individual NPs subject to local variations and thereof, time is an important parameter. In order to prove this in [35], we measured citrate-capped Au and AgNPs diluted with ultrapure water (1/4) of different sizes at different times after their dilution (up to 72 h). Fractograms showed that there was a decrease in the absorbance signal with time due to dissolution of NPs, and that decrease depended on the type of NP and its size, being lower for Au and for 20 nm core size for both types of NPs.

**Figure 2:**
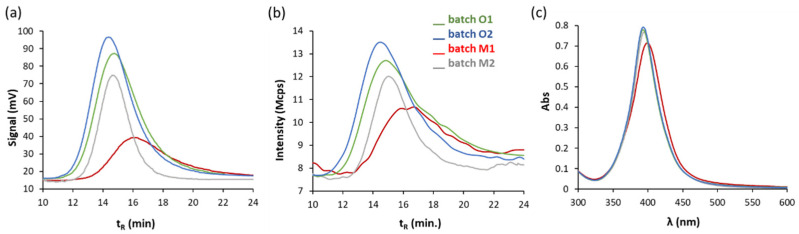
Fractograms obtained with (**a**) UV-Vis and (**b**) DLS detectors, and (**c**) UV-Vis spectra of dispersions diluted 1/4 with ultrapure water (5 mg·L^−1^) of various commercial batches of AgNPs.

Four different batches of commercial NPs were tested here: M1, M2, O1 and O2 (see the previous section for explanation). The spectra obtained for diluted dispersion with ultrapure water before 2 h of preparation were the same for each batch assayed. Figure 2 includes the fractograms and UV-Vis spectra corresponding to fresh dispersions diluted 1/4 with ultrapure water. Fractograms recorded using UV-Vis detector (Figure 2a) show a clear difference in batch M1 in reference to the others, in which a displacement of the maximum peak is observed up to 16 min, which is consistent with the presence of larger NPs, as well as a decrease in the signal at λ = 395 nm, which can respond to an SPB sift to higher wavelengths than those corresponding to the other dispersions as Table 2 shows, but also due to lower mass of AgNPs. The peak areas were 27.9, 25.9, 23.3 and 22.9 V.s for batches O2, O1, M2 and M1, respectively.

The fractograms recorded using the DLS detector indicate less monodispersity for batch M1 with respect to the rest (see Figure 2b). In addition, batch mode DLS data show a mean hydrodynamic diameter around 2 nm larger than the others, as shown in Table 2. All batches have a similar SPB by means of UV-Vis spectroscopy, although batch M1 shows some differences, since it provides a somewhat lower absorption and a slight bathochromic shift, which can be due to AgNPs with a slightly larger size (Figure 2c)

Phenomena such as dissolution and/or passivation can be considered as a consequence of prolonged exposure to atmospheric conditions of the batch dispersion M1 [35,38,39,40,41]. So that a part of AgNPs of the dispersion can be dissolved and released into the environment as Ag^+^, or it can be oxidized, generating an Ag_2_O surface film, which was indicated in other works by means of a bathochromic shift of the plasmonic band, as well as a broadening and a lower height (see Table 2 and Figure 2c). If AF4 records are compared with those obtained by UV-Vis spectroscopy, it should be noted that AF4 technique shows more differences besides giving their size. From the characterization results of the several batches, we selected O1 and O2 for studying the performance of the bulk NPs by diluting with several environmental water samples.

### 3.2. Behaviour of the Dispersions in Function of Environmental Water Used as Diluent

The behavior of AgNPs in matrices such as ultrapure, bottled, tap, transitional and sea waters were studied. Table 3 indicates pH, electrical conductivity and redox potential values of the several water matrices, showing significant variations in the measured parameters as expected. Besides the ʒ-potentials measured by off-line DLS of 1:4 diluted dispersions of citrate capped AgNPs in the water matrices after 1 min of their mixtures are given. Similar ʒ-potential values were obtained for both batches, M and O. The most negative ʒ- potentials were achieved by dilution with ultrapure water (the most stable dispersions) and the least one with sea water. Drinking waters presented values between −14.06 and −17.67 mV. Transitional and sea waters gave the lesser values.

**Figure 3:**
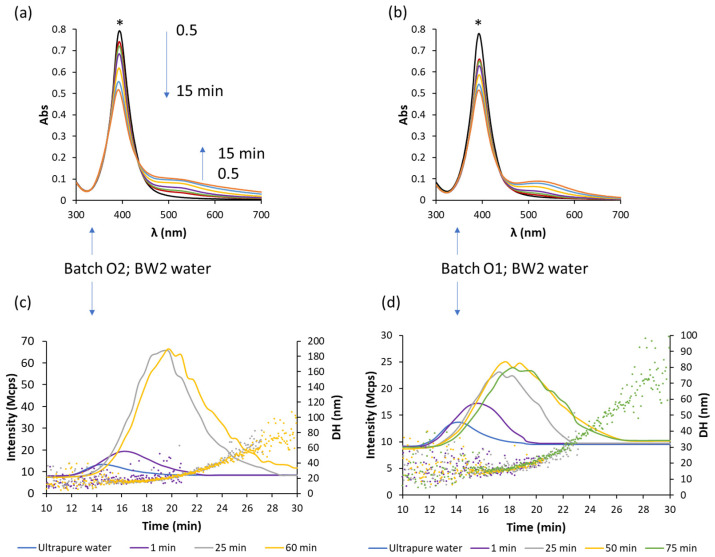
Spectra of batches O2 (**a**) and O1 (**b**) diluted 1/4 with bottled mineral water measured at several times (0.5, 1, 2, 5, 10, 15 min, the SPR band for the dilution with ultrapure water was also included, marked with *) and their fractograms (solid lines) (**c**) and (**d**), respectively, by using dynamic light scattering (DLS) and hydrodynamic diameter (points) measured at different times.

Figure 3a,b compares the spectra measured up to 15 min of two diluted dispersions (O1 and O2) with bottled water (BW2) and the fingerprint was different to that obtained with ultrapure water, for this last matrix stability at the assayed times was observed. Note that the ʒ-potential for ultrapure water-diluted dispersions is more negative than the value achieved with BW2 as Table 3 shows. The obtained registers are compatible with the aggregation of AgNPs in BW2. Fractograms obtained by using the DLS detector given in Figure 3c,d for these dispersions show an increase in the size with respect to that obtained with ultrapure water injected in the AF4 at 1 min of their mixture. For longer times (up to 75 min) the sizes increased markedly as Figure 3c,d indicated for both dispersions, although the evolution fingerprints were different between both dispersions.

**Figure 4:**
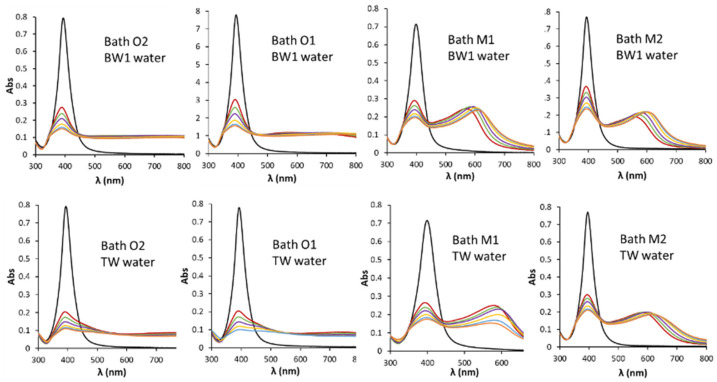
Spectra of the several batches diluted 1/4 with bottled mineral water 1 and tap water measured at several times (0.5, 1, 2, 5, 10, 15 min). The SPR band for the dilution with ultrapure water was also included.

Decreasing of the plasmonic band can be attributed to a concentration decrease of the initial AgNPs as a consequence of changes in their electrostatic environment, which lead to processes like aggregation, which cause structural, morphological and surface alterations. In this sense, as can be seen in Figure 4, a higher degradation rate is observed when BW1 and TW waters were employed for diluting batches O instead of BW2 water (see Figure 3). The profiles obtained were similar for the batches with the same caducity data. Those waters presented higher conductivity than BW2 as can be seen in Table 3, and then, higher ionic strength. The aggregation is more severe for O1 and O2 batches than that obtained for M1 and M2 batches in BW1 and TW waters, although their ʒ-potentials were somewhat greater in absolute values for O than for M batches. The electrostatic interactions between a charged NP and an electrolytic solvent fall within one of three regimes as proposed in [37] for citrate capped AuNPs, according to the net charge of the sphere: (i) depolarized (σ = surface charge density < ~0.6 e nm^−2^), where the thermal motion of the sodium counterions overcomes the electrostatic attraction toward the NP; (ii) mildly polarized (~0.6 < σ < ~4.1 e nm^−2^), in which the Coulomb forces attract enough ions into the Stern layer to screen out the charge load of the NP, and (iii) hyperpolarized (σ > ~4.1 e nm^−2^), the situation at which the counterions’ steric and electrostatic hindrance, as well as their loss of translational degrees of freedom, limit their binding onto the NP. The chemisorption of citrate onto the assembled metallic surfaces critically depends on variables such as the particle size, the surface charge density, and the ionic strength of the medium in which the NPs are dispersed [37].

**Figure 5:**
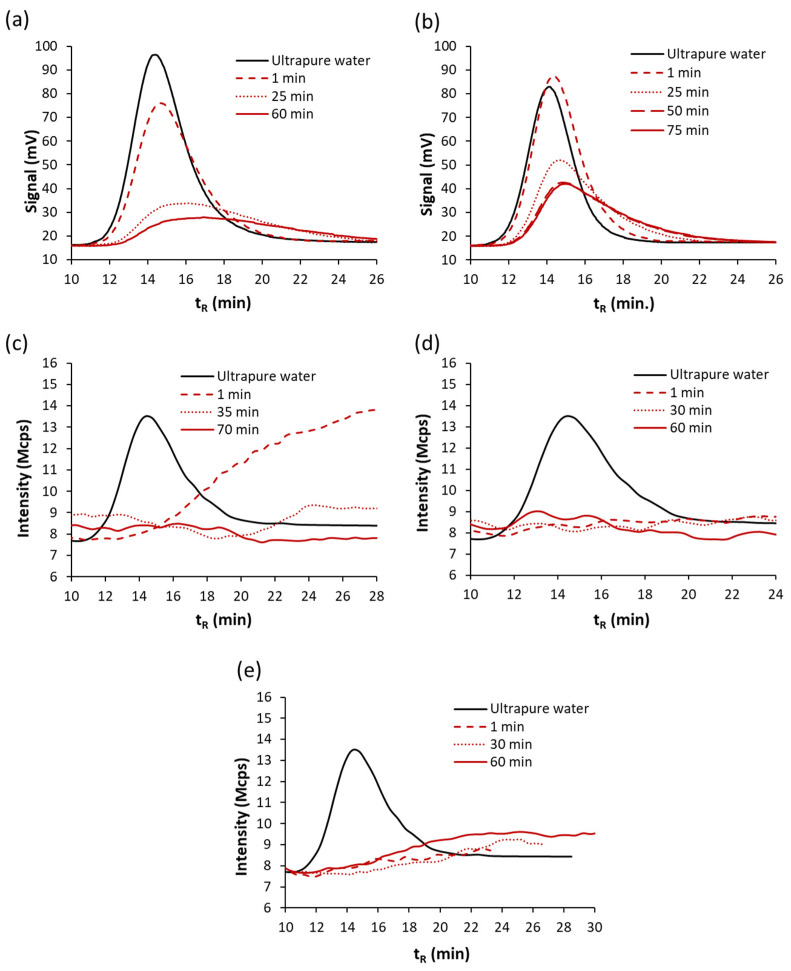
Fractograms (λ = 395 nm) obtained for suspensions diluted 1/4 of batches O2 (**a**) and O1 (**b**) with BW2 water at different times and batch O2 with water bottled water (BW1) (**c**), tap water (TW) (**d**) and sea water (SW) (**e**) at different times using the DLS detector.

Figure 5 shows the fractograms obtained by using UV-Vis and DLS detectors in series for suspensions of studied batches diluted with BW2, BW1 and TW water matrices. Profiles for O1 and O2 batches and BW2 are in accordance with those obtained by DLS detection given in Figure 3c,d for this matrix, which may be due to changes of AgNPs in their size due to aggregation, a bathochromic shift can generate an absorbance decrease at the observed wavelength (395 nm). An increase in retention times and a peak broadening are also observed, which indicate the formation of larger AgNPs. AF4 also shows that the kinetics of the process is bigger for batch O2; batch O1 for 1 min gave a similar signal to UPW dilution. Those results are in accordance with spectra obtained at 1 min (see Figure 3a,b). Fractograms for higher times for batch O1, show polydispersity too, but in less extension than that provided by batch O2.

Batch O2 diluted in BW1 water, with a higher electrolyte content than BW2, measured after 1 min of preparation by using DLS detector showed a distribution of NPs in a wide range of sizes; besides no significative UV-Vis signal was obtained at 395 nm, which can be due to aggregation of almost all of the initial AgNPs (see Figure 5c). These results are in accordance with those shown in Figure 4, obtained by UV-Vis spectroscopy. Suspensions with a high fraction of aggregated AgNPs give rise to fractograms in which the UV-Vis signal is very weak, being indistinguishable from the baseline working at a cross-flow of 1 mL.min^−1^ (see Table 1).

Suspensions prepared with waters with higher ionic strength (BW1 and TW) show no significate signal with respect to baseline for both detectors at assayed times, as can be seen in Figure 5c,d for the DLS detector. The loss of signal can be related to the aggregation of AgNPs with higher sizes than those presented by BW2. All of this seems to show a trend of size transformation of AgNPs with the ionic strength of the medium. NP aggregation can be induced by increasing the salt concentration of the medium, in [37] it is found that alterations in the aggregation state of the NPs arise mostly from the screening of the NP charge by the sodium counterions in solution. These ions can rest at the interface of the metallic bodies and form salt bridges that stabilize NP–NP dimer.

**Figure 6:**
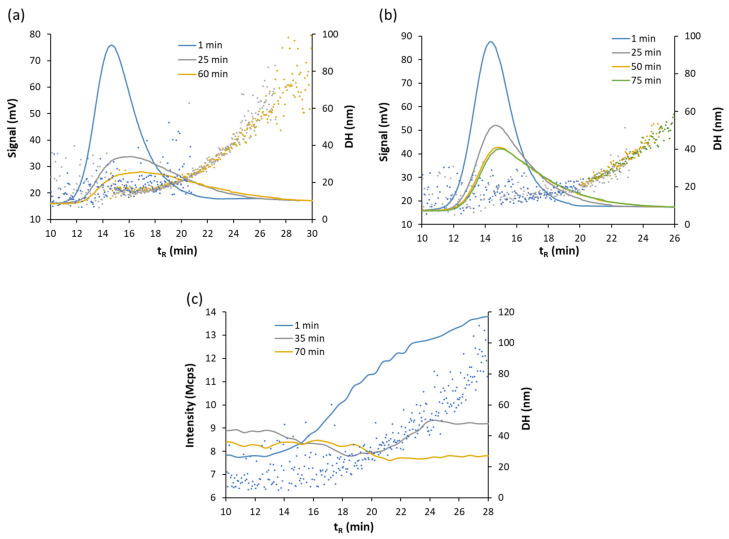
Fractograms (solid line) and hydrodynamic diameter (points) obtained for suspension of batches O2 (**a**) and O1 (**b**) diluted 1/4 with BW2 water at different times using UV-Vis detector. (**c**) Fractograms and hydrodynamic diameter for suspension of batch O2 diluted 1/4 with BW1 water at different times using the DLS signal.

Figure 6 includes the hydrodynamic diameter estimated by the DLS detector along the fractograms using AF4-UV-Vis-DLS for BW1 and BW2 dilutions. It can be observed that for batch O2, the use of bottled water of lower hardness (BW2, 13 and 28 mg/L of Mg and Ca, respectively) for dilution, Figure 6a, provided less variation in NP sizes than in the case of harder bottled water (BW1, 35 and 55 mg/L of Mg and Ca, respectively), Figure 6c, a trend that has also been observed in various studies using other analytical techniques [8,42,43]. Batch O1, Figure 6b, provided less variation of sizes than batch O2 when BW2 water was used.

AF4-UV-Vis-DLS aids to understand the magnitude and nature of changes that aqueous dispersant composition can produce in colloidal systems. All these phenomena are characteristic of DLVO-type behavior [39,42,43], where, according to this theory, there is a minimum concentration of each of the saline species from which there is a regime of rapid aggregation or diffusion-controlled aggregation, which is called critical coagulation concentration (CCC) [44,45].

In this sense, the electrolytic substances present in the bulk of AgNP aqueous dispersions produce a shielding of the charge that covers them, reducing their protection against aggregation processes. If the concentration of these species is below the CCC, aggregation takes place through slow kinetics, a situation known as reaction-limited aggregation regime, in which NPs are partially kinetically stabilized by the repulsion of the electrical double layer, as seems to be observed in suspensions prepared with BW2 water. Conversely, if electrolyte concentration is higher than the CCC, aggregation occurs more quickly as for BW1 and TW (43 and 115 mg/L of Mg and Ca, respectively), a situation known as diffusion-controlled aggregation regime, in which the charge that covers the NPs is sufficiently shielded and the energy barrier is insufficient to prevent the aggregation process [39,42,43].

**Figure 7:**
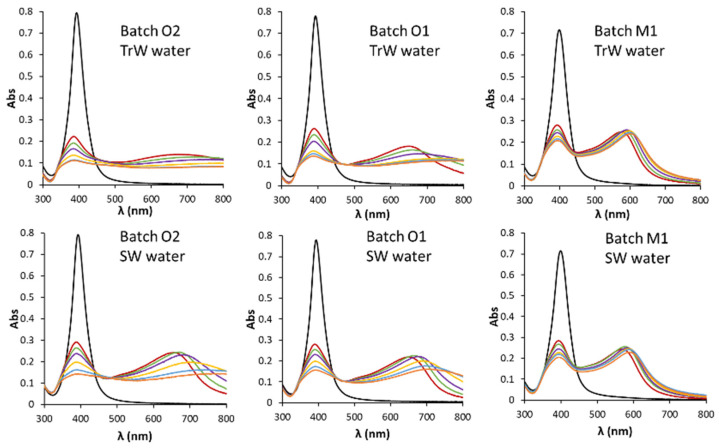
Spectra of the several batches diluted 1/4 with transitional and sea waters measured at several times (0.5, 1, 2, 5, 10, 15 min). The SPR band for the dilution with ultrapure water was also included.

Since the mechanism through which electrolytes facilitate aggregation consists in the shielding of the charge that covers the NPs, some factors such as ion charge are directly related to their shielding capacity, and consequently, induce the aggregation process. In this sense, there is an inverse dependence of the CCC with the sixth power of the ion charge [43] giving rise to a greater influence of divalent ions compared to monovalent ions between 50–83 times [42] in the aggregation process. Their stability depends to a great extent on the stabilizing agent that is part of their coating. In this sense, the characteristics and behaviors observed are attributable to the electrostatic stabilization mechanism, since AgNPs stabilized by steric effect by coatings such as PVP, PEG or branched polyethyleneimine (BPEI) do not show DLVO-type behavior and are stable in suspensions in the presence of electrolytes with various units of molar concentration [8,43]. Therefore, in drinking water, the aggregation process should be modulated mainly by concentration of Ca^2+^ and Mg^2+^, and definitely, by water hardness. On the other hand, in transitional and sea waters (see Figure 7), given the highest concentration of dissolved ionic substances, the stability of AgNPs should be strongly restricted. However, Figure 7 shows that the aggregation kinetics is faster for TrW than for SW (see also Figure 5e). Moreover, for batches O the rate order corresponded to BW2, SW, TrW, BW1 and TW, being BW2 the lowest and TW the highest. The same order can be established for batch M, but with slower kinetics. AF4 results supported the data obtained by spectroscopy.

TrW and SW presented a lower speed with respect to the expected one in comparison with other aqueous matrices with much lower ionic strength (BW1 and TW, Figure 4) and higher ʒ-potentials in absolute values (see Table 3), which does not seem to be in agreement with the dependence of this speed with the ionic strength of the medium previously observed and the ʒ-potentials. These environmental waters are complex media in which other factors can also play a role in AgNP stability. In this sense, various works show the stabilizing effect of dissolved organic matter (DOM), mainly fulvic and humic acids, which seems to form AgNP–OM complexes that provide a new external coating in the form of an adsorbed layer that acts as a stabilizer by steric effect [42,46,47,48,49]. Additionally, the high concentration of halides, mainly chlorides, can cause the passivation of AgNPs forming a superficial layer of AgX [36,38,42].

Definitely, AgNPs behavior in aqueous matrices and the processes and transformations in which they intervene depends on a large number of factors, especially in natural waters. Some such as ionic strength, the effect of different cations and anions, organic matter and dissolved O2 were discussed. However, natural aquatic environments are much more complex, and many other factors such as interaction with natural colloids, heteroaggregation or the presence of large particulate matter are still poorly understood and require great difficulty in undertaking their study.

### 3.3. Characterizing Information: AF4 vs. Static DLS and UV-Vis Spectroscopy

**Figure 8:**
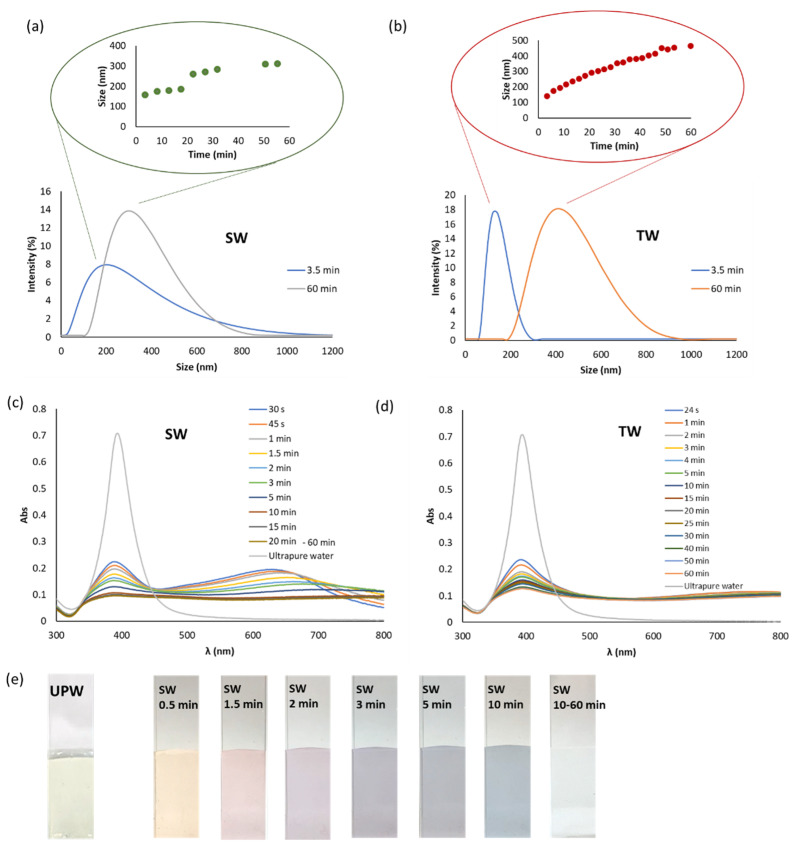
Static DLS and UV-spectroscopy results for batch O2 diluted 1/4 with sea (SW) and tap (TW) waters measured at several times: (**a**) DLS registers for SW at different times including an insert, which shows the changes in size of AgNPs with time ; (**b**) DLS registers for TW at different times including an insert, which shows the changes in size of AgNPs with time; (**c**) Spectra of AgNPs with SW at several times, the SPR band for the dilution with ultrapure water was also included; (**d**) Spectra of AgNPs with TW at several times, the SPR band for the dilution with ultrapure water was also included; (**e**) Colour of the suspension in SW with time.

This section was focused to determine the information given by AF4 in reference to batch mode DLS and UV-Vis spectroscopy. We selected TW and SW waters, which provide different fingerprints as can be seen in the previous section. Figure 8 summarizes the results given by off-line DLS and UV-vis spectroscopy for batch O2. These off-line DLS results (Figure 8a,b) show that the size of the aggregated AgNPs increased with time for both types of waters and also their polydispersity, more markedly for TW in accordance with that established in the previous section. The aggregation fingerprint was faster for TW than for SW, this last water presented different colors as can be seen in Figure 8e in the function of time. However, TW dispersions were colorless.

**Figure 9:**
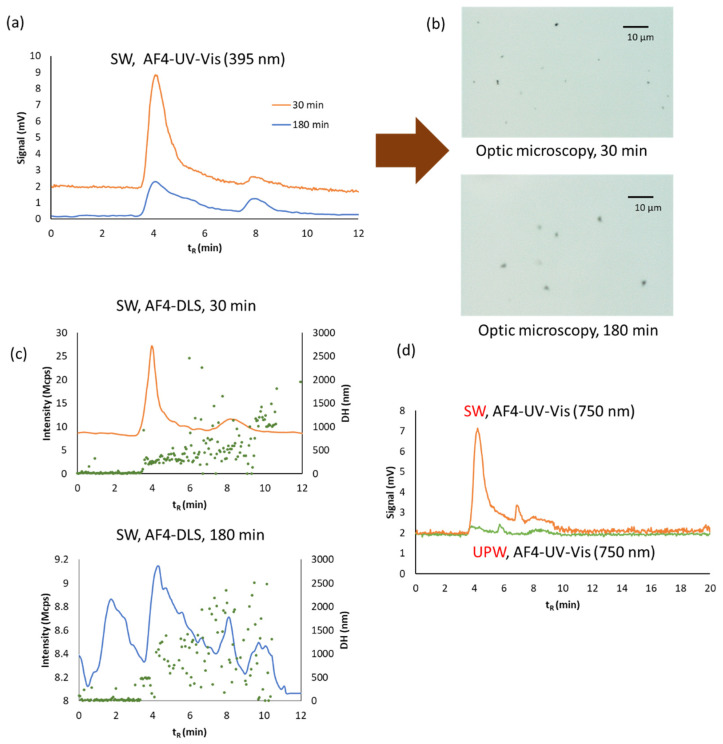
AF4 coupled in series with UV-Vis and DLS detectors for batch O2 diluted 1/4 with sea (SW) water measured at several times: (**a**) fractograms at 395 nm; (**b**) optic images at 30 and 180 min; (**c**) DLS fractograms at 30 and 180 min: (**d**) Fractograms at 750 nm of SW and UPW at 30 min. Crossflow was 0.2 mL·min^−1^.

**Figure 10:**
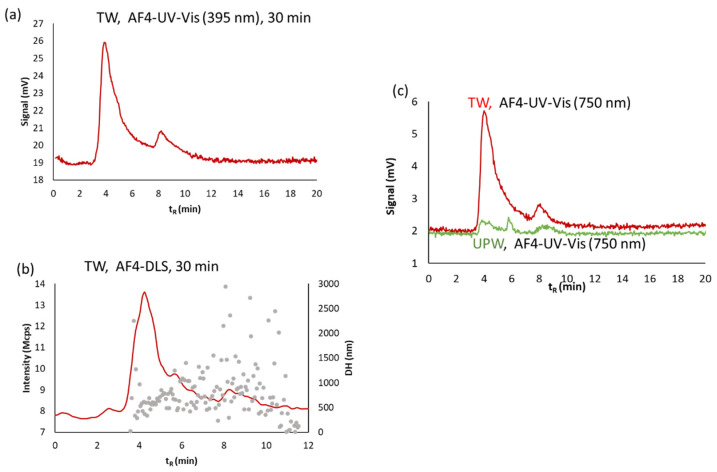
AF4 coupled in series with UV-Vis and DLS detectors for batch O2 diluted 1/4 with tap (TW) water measured at 30 min: (**a**) fractograms at 395 nm; (**b**) DLS fractograms; (**c**) Fractograms at 750 nm of TW and UPW. Crossflow was 0.2 mL·min^−1^.

In order to study the polydispersity of the aggregated AgNPs, a slow crossflow was assayed (0.2 instead 1.0 mL·min^−1^ given in Table 1) for AF4. When lower crossflow was employed the retention times for bigger aggregates diminishes. Fractograms obtained for dispersions in SW and TW waters are given in Figure 9 and Figure 10, respectively. AF4 with UV-Vis and DLS in series showed two populations of NPs with different sizes for both dispersants. Dispersions in SW at 30 and 180 min measuring at 395 nm presented two peaks indicating two populations with different sizes (Figure 9a). Images obtained by optical microscopy showed the greater size of both aggregates at 180 min than those obtained at 30 min (Figure 9b). DLS detector permits the evaluation of the sizes as it can be seen in Figure 9c, fractograms indicate that the aggregates are bigger at 180 min than those obtained at 30 min. Figure 9d shows the fractograms at 750 nm giving the two populations too. A baseline signal was obtained for UPW dispersion at 750 nm, which is in accordance with its color (see Figure 8e) indicating no aggregation.

Considering the behavior of AgNPs in TW, two populations were also obtained by AF4 (see Figure 9). The sizes of the two colorless aggregates at 30 min were higher than those obtained in SW (see Figure 10). These results are in agreement with the aggregation rate described in the previous section and observed by UV-Vis spectroscopy in Figure 8.

It is shown that AF4 coupled online with UV-Vis and DLS in series gives relevant information for characterizing the aggregation processes of AgNPs in the different water matrices with respect to static DLS and UV-Vis spectroscopy.

## 4. Conclusions

Diluted-dispersions of citrate capped AgNPs with ultrapure, several drinking, transitional and sea waters were studied for short times (up to 180 min). The water matrix influenced AgNP stability and it was demonstrated that aggregation was the process involved. Aggregation kinetics depended on the type of water. Several sizes were obtained in the function of the water, which determined the color of AgNPs.

Definitely, AgNP behavior in aqueous matrices and the processes and transformations in which they intervene depends on a large number of factors. Some such as ionic strength, the effect of different cations and anions, organic matter, natural colloids, heteroaggregation, among others, can justify their different performance. Ultrapure water conserved citrate capping to a great extent and then, stability of NPs. Drinking waters with similar ʒ-potentials, responded to ionic strength in the aggregation process. Transitional and sea waters presented more stability than expected, probably due to steric effects of dissolved organic matter or passivation due to the high level of chloride.

It was demonstrated that AF4 coupled online with UV-Vis and DLS in series provides relevant information for characterizing the aggregation processes of AgNPs in the different water matrices studied with respect to static DLS and UV-Vis spectroscopy. Several populations of aggregates were obtained by AF4, which provides new information and also permits the interpretation of the results achieved by static DLS and UV-Vis spectroscopy. The use of the proposed technique provides valuable information for NP performance in the environment.

## Figures and Tables

**Table 1:** Flow program used for silver nanoparticle (AgNP) analysis by AF4. Detection flow: 0.5 mL·min^−1^. * For assays to study the polydispersity of the aggregated AgNPs. For more explanations see text.

	Time, min	Direct Flow, mL·min^−1^	Crossflow, mL·min^−1^	Focus Flow, mL·min^−1^
Focus	7	0.2	1.0 (0.2) *	1.3
Transitional	0.5	0.2 to 1.5	1.0 (0.2) *	1.3 to 0
Fractionation	35	1.5 to 0.5	1.0 (0.2) * to 0	0
	10	0.5	0	0

**Table 2:** Wavelength values (λ) of SPB, width of the peak at half the maximum, extinction coefficient (ε_máx_) obtained by UV-Vis spectroscopy and hydrodynamic diameter (DH) for different commercial batches of AgNPs.

Batch	λ SPB (nm)	Width SPB_1/2_ (nm)	ε_máx_ (mM^−1^·cm^−1^)	DH (nm)
M1	398 ± 2	50.2 ± 0.1	15.43 ± 0.02	22 ± 2
M2	396 ± 1	42.9 ± 0.1	16.58 ± 0.02	22 ± 3
O1	393 ± 2	42.7 ± 0.2	16.83 ± 0.05	21 ± 3
O2	393 ± 2	42.8 ± 0.2	17.05 ± 0.03	20 ± 3

**Table 3:** pH, electrical conductivity (EC) and redox potential for each aqueous matrices used. ʒ-potentials for M and O batches of AgNPs diluted 1:4 with several water matrices (see the experimental section for more explanation.)

Water Matrix	Abbreviation	pH	CE (μS·cm^−1^)	Potential (mV)	ʒ-Potential of AgNPs: M/O (mV)
Ultrapure water	UPW	6.0 ± 0.2	6.2 ± 0.1	250 ± 10	**−41.27/−42.97**
Bottled water	BW2	7.37 ± 0.15	272 ± 4	190 ± 30	**−15.87/−17.67**
Bottled water	BW1	7.52 ± 0.04	570 ± 30	300 ± 60	**−14.06/−14.83**
Tap water	TW	7.39 ± 0.18	10903 ± 40	630 ± 30	**−14.36/−14.76**
Transitional water	TrW	7.67 ± 0.17	(13.4 ± 0.2) × 10^3^	220 ± 60	**−8.86/−10.41**
Sea water	SW	7.99 ± 0.07	(58 ± 3) × 10^3^	150 ± 30	**−5.42/−5.92**
